# Robustness of a convolutional neural network trained on dermoscopic images and challenged with close‐up images

**DOI:** 10.1111/ddg.15900

**Published:** 2025-10-11

**Authors:** Anastasia Sophie Vollmer, Julia Katharina Winkler, Katharina Susanne Kommoss, Wilhelm Stolz, Albert Rosenberger, Alexander Enk, Holger Andreas Haenssle

**Affiliations:** ^1^ Department of Dermatology University medical Center Heidelberg Heidelberg Germany; ^2^ Department of Dermatology Allergology and Environmental Medicine II Hospital Thalkirchner Street Munich Germany; ^3^ Department of Genetic Epidemiology University Medical Center Georg‐August University of Goettingen Goettingen Germany

**Keywords:** Clinical images, dermoscopy, DL‐CNN, robustness, teledermatology

## Abstract

**Background and Objectives:**

Deep learning‐convolutional neural networks (DL‐CNNs) have demonstrated high diagnostic accuracy within the domain of dermoscopy. However, many clinical settings lack dermoscopic devices, requiring reliance on close‐up images. This study evaluates the robustness of a DL‐CNN trained on dermoscopic images when challenged with close‐up images.

**Methods:**

In this cross‐sectional study, the DL‐CNN Moleanalyzer pro, trained on 129,487 dermoscopic images, was tested on 350 skin lesions, each imaged both clinically and dermoscopically. Histopathology (89.4%) or expert consensus with a two‐year follow‐up (10.6%) served as the reference standard. Primary outcomes included sensitivity, specificity, and the receiver operating characteristic‐area under the curve (ROC‐AUC).

**Results:**

For dermoscopic images, the DL‐CNN achieved a sensitivity of 88.2% (95% CI: 82.9%–92.0%), specificity of 69.0% (61.4%–75.8%), and a ROC‐AUC of 0.866 (0.860–0.873). For close‐up images of the same lesions, sensitivity decreased to 60.5% (53.5%–67.1%, *p* < 0.001), while specificity increased to 79.4% (72.3%–85.0%, *p* = 0.027). The ROC‐AUC for close‐up images was 0.780 (0.772–0.790, *p* = 0.003).

**Conclusions:**

Our findings highlight the diagnostic limitations of applying DL‐CNNs trained on dermoscopic images to close‐up images, with reduced sensitivity but increased specificity. The results emphasize the need to improve cross‐domain adaptability. Fine‐tuning of DL‐CNNs on clinical images may enhance real‐world diagnostic accuracy.

## INTRODUCTION

Cutaneous malignancies are among the most common neoplasms in Western populations, accounting for a substantial global health burden.^1^ This challenge is anticipated to increase due to the ongoing demographic shift toward an aging population.^1^, ^2^ While dermoscopy greatly enhances diagnostic accuracy in skin cancer screening, particularly for pigmented lesions,^3^, ^4^, ^5^ its accessibility remains limited in primary care, teledermatology, and mobile applications for patient self‐examination.^6^ Clinical close‐up images, which are easier to acquire, represent a practical alternative to dermoscopic images.

At the same time, deep learning‐convolutional neural networks (DL‐CNNs) are emerging as promising tools for skin cancer diagnostics, especially in the context of human‐machine collaboration.^7^, ^8^ Despite demonstrating high diagnostic accuracy when trained and tested on dermoscopic images, especially for melanocytic lesions,^7^, ^8^, ^9^, ^10^, ^11^, ^12^, ^13^ the translation of DL‐CNN performance from controlled research settings to clinical practice remains challenging.^6^ Computer vision systems may lack the robustness of human vision, particularly when faced with minor variations in imaging conditions and skin types.^14^, ^15^ Specifically, convolutional architectures were shown susceptible to image perturbations such as translations or rescaling.^14^, ^16^ Although training data augmentation can partially address these limitations, its effectiveness is limited for images that differ significantly from the training set.^16^, ^17^, ^18^

Nevertheless, the actual cross‐domain robustness of market‐approved DL‐CNNs, which have generally been trained on dermoscopic data, is still largely unexplored. Importantly, many of these models may not yet utilize the state‐of‐the‐art hybrid artificial intelligence (AI) architectures currently tested in research.^19^, ^20^, ^21^ Our study presents a critical head‐to‐head comparison of the binary (benign/malignant) classification performance of a market‐approved DL‐CNN using matched clinical close‐up and dermoscopic images of the same lesions. This approach allows to highlight differences in sensitivity and specificity, providing valuable insights into the real‐world applicability of DL‐CNNs across various imaging modalities. The findings may contribute to improving the robustness of DL‐CNNs for clinical use, particularly in applications such as whole‐body imaging, teledermatology, AI‐powered smartphone tools, web‐based diagnostic services, and skin screenings conducted in primary care settings.^6^, ^22^, ^23^, ^24^

## MATERIAL AND METHODS

This cross‐sectional study, approved by the local ethics committee (S‐629/2017) and conducted in accordance with the *Declaration of Helsinki*, utilized a binary DL‐CNN (Moleanalyzer pro, FotoFinder Systems Inc.) based on Google's Inception_v4 architecture. The DL‐CNN was trained on 129,487 dermoscopic images (100,021 benign, 29,466 malignant lesions). Further details are available in the online supplementary Methods section (M1). A malignancy score ranging from 0–1 was generated (> 0.5 indicating malignancy). As part of DL‐CNN preprocessing, images were resized to 299 × 299 pixels to ensure standardized input dimensions across all image types. Clinical close‐ups were cropped to reduce confounders such as skin markings^25^ and vignetting artefacts^26^ (online supplementary Figure S1).

### Test‐set

**FIGURE 1:**
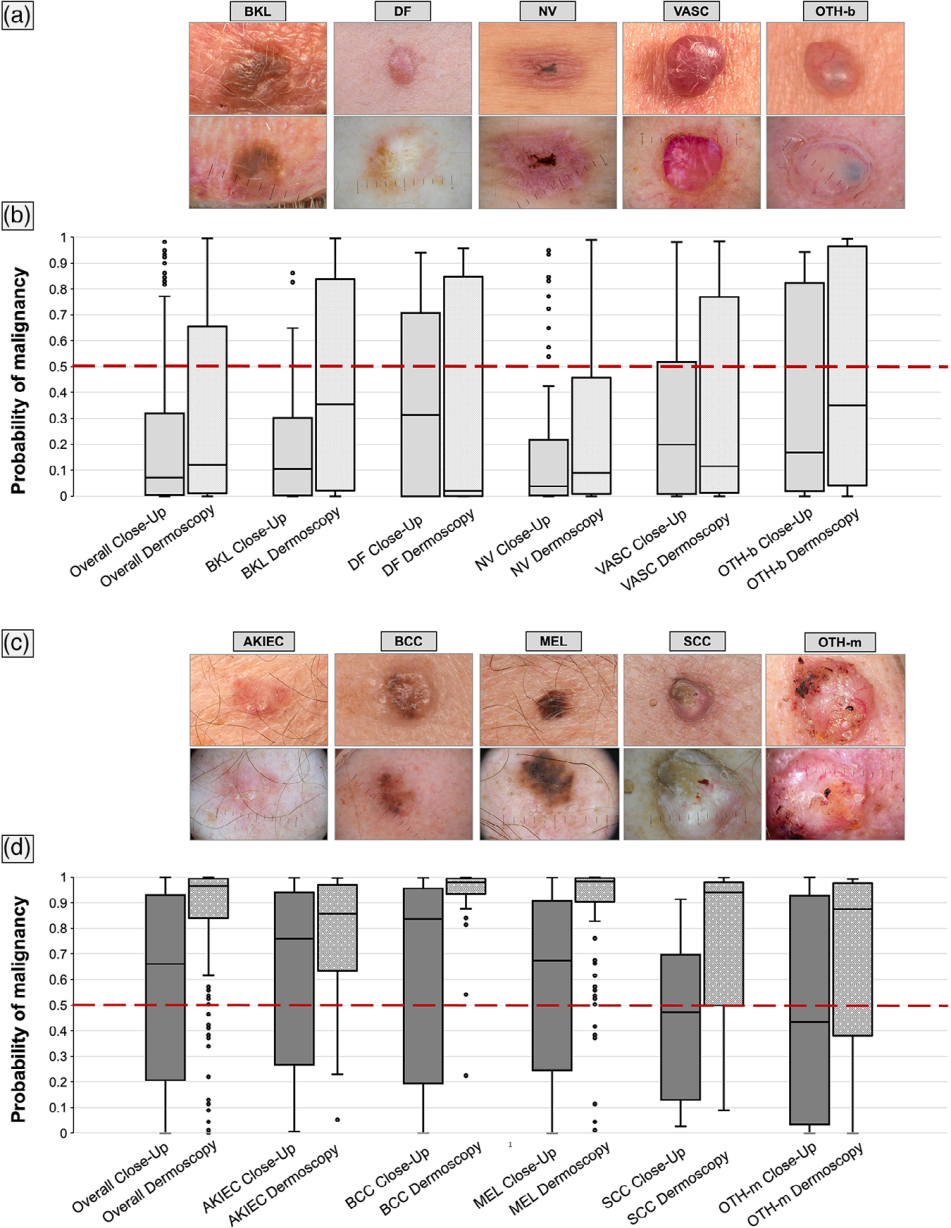
Representative close‐up images with corresponding dermoscopic images below for benign (a) and malignant (c) lesions. (a) Benign diagnostic classes from left to right: solar lentigo and seborrheic keratosis (BKL; such as seborrheic keratosis), dermatofibroma (DF; such as dermatofibroma), nevi (NV; such as recurrent nevus), benign vascular neoplasms including angioma and pyogenic granuloma (VASC; such as hemangioma), and other benign lesions such as spiradenoma (OTH‐b). (c) Premalignant or malignant diagnostic classes from left to right: actinic keratosis/Bowen disease (AKIEC; such as actinic keratosis), basal cell carcinoma (BCC; such as pigmented BCC), melanoma (MEL; such as melanoma in situ), squamous cell carcinoma including Bowen carcinoma (SCC; such as SCC), and other malignant lesions such as melanoma metastasis (OTH‐m; such as cutaneous breast cancer metastasis). (b, d) DL‐CNN malignancy scores (ranging from 0 to 1, with a malignancy cut‐off > 0.5) for benign (b) and malignant (d) diagnostic classes are shown as boxplots. The boxplots on the left display DL‐CNN malignancy scores for close‐up images, while those on the right show scores for dermoscopic images. Higher scores (closer to 1) indicate a greater likelihood of malignancy. The cut‐off for malignancy (> 0.5) is indicated by a red dotted line. The boxes represent the interquartile range (25th–75th percentiles), with the median depicted as the horizontal line within the box. Whiskers extend to the minimum and maximum scores within the data range.

A conveniance sample of 350 skin lesions (195 malignant, 155 benign) was drawn from the image database at the University Department of Dermatology, Heidelberg, by selecting cases from various diagnostic classes imaged during routine clinical care between 2014 and 2024. No overlap between training and test sets was allowed. Each lesion was imaged both clinically (close‐up) and dermoscopically. Image acquisition was performed using various camera and dermatoscope setups with polarized light. Out of 350 clinical images, 39 were taken using a smartphone. A total of 321 (91.7%) diagnoses were confirmed by histopathology, and 29 (8.3%) diagnoses of non‐excised lesions through expert consensus (formal agreement among 3 experienced specialists) in combination with an uneventful follow‐up period of at least 2 years, which applied to benign lesions. The dataset included pigmented and non‐pigmented lesions from various body sites (Table 1). Benign cases included benign keratinocytic lesions such as solar lentigo and seborrheic keratosis (BKL), dermatofibroma (DF), nevi (NV), benign vascular neoplasms including angioma and pyogenic granuloma (VASC), and other benign lesions such as spiradenoma (OTH‐b) (Table 1, Figure 1a). Premalignant or malignant cases comprised actinic keratosis/Bowen disease (AKIEC), basal cell carcinoma (BCC), melanoma (MEL), squamous cell carcinoma including Bowen's carcinoma (SCC), and other malignant lesions such as melanoma metastasis (OTH‐m) (Table 1, Figure 1c).

**TABLE 1:** Characteristics of skin lesions in the test set (n = 350).

	Benign lesions (n = 155)		Malignant lesions (n = 195)	

	*n*	%	*n*	%
***Localization***				
Scalp	11	7.1	10	5.1
Face/Lip	34	21.9	61	31.3
Genital Organ	–	–	1	0.5
Trunk	64	41.3	87	44.6
Extremities	35	22.6	28	14.4
Acral Unit	11	7.1	8	4.1
***Ground truth***				
Histopathology	127	81.9	194	99.5
Expert consensus (EC) and unremarkable follow‐up (FU) (> 2 years)	28	18.1	1^*^	0.5
***AKIEC***			**15**	**7.7**
Actinic keratosis			8	4.1
Bowen Disease			7	3.6
*Histopathology*			14/15	93.3
***BCC***			**35**	**18**
Fibroepithelioma of Pinkus			1	0.5
BCC			32	16.4
Collision BCC and angioma			1	0.5
Collision BCC and nevus			1	0.5
*Histopathology*			35/35	100
***MEL***			**106**	**54.4**
In situ melanoma				
Melanoma in situ			8	4.1
Lentigo maligna			10	5.1
Invasive melanoma				
Lentigo maligna melanoma			7	3.6
amelanotic			2	1
nodular			11	5.6
acrolentiginous melanoma			4	2.1
spitzoid			6	3.1
desmoplastic			1	0.5
superficial spreading			25	12.8
not specified in histologic report			32	16.4
*Histopathology*			106/106	100
***SCC***			**6**	**3.1**
SCC			5	2.6
Bowen's carcinoma			1	0.5
*Histopathology*			6/6	100
***OTH‐m***			**33**	**16.9**
Metastasis				
from pulmonary carcinoma			3	1.5
from breast cancer			1	0.5
from melanoma			19	9.7
Atypical fibroxanthoma			2	1
PDS			3	1.5
Mycosis fungoides			1	0.5
Merkel cell carcinoma			3	1.5
Kaposi's sarcoma			1	0.5
*Histopathology*			33/33	100
***BKL***	**27**	**17.4**		
Seborrheic keratosis	21	13.6		
Solar lentigo	6	3.9		
*Histopathology*	25/27	92.6		
***DF***	**6**	**3.9**		
*Histopathology*	5/6	83.3		
***NV***	77	49.7		
Blue nevus	11	7.1		
Spitz nevus	5	3.2		
Dysplastic nevus	12	7.7		
Recurrent nevus	3	1.9		
Plantar nevus	1	0.7		
Becker's nevus	1	0.7		
Compound nevus	15	9.7		
Papillomatous nevus	4	2.6		
Combined nevus	6	3.9		
Mechanically irritated nevus	6	3.9		
Congenital nevus	13	8.4		
*Histopathology*	56/77	72.7		
***VASC***	27	17.4		
Hemangioma	18	11.6		
Angiofibroma	1	0.7		
Granuloma pyogenicum	5	3.2		
Angiokeratoma circumscriptum	1	0.7		
Angiokeratoma	2	1.3		
*Histopathology*	25/27	92.6		
***OTH‐b***	18	11.6		
Spiradenoma	2	1.3		
Cutaneous cylindroma	1	0.7		
Poroid hidradenoma	1	0.7		
Cutaneus pseudolymphoma	1	0.7		
Clear cell acanthoma	2	1.3		
Plantar collagenoma in Proteus syndrome	1	0.7		
Condyloma acuminatum	1	0.7		
Juvenile xanthogranuloma	1	0.7		
Nevus sebaceus	1	0.7		
Sebaceous adenoma	4	2.6		
Trichofollikuloma	1	0.7		
Trichoblastoma	1	0.7		
Verruca vulgaris	1	0.7		
Histopathology	16/18	88.9		

* Clinically diagnosed actinic keratosis

*Abbr*.: AKIEK, actinic keratosis/Bowen disease; BCC, basal cell carcinoma; BKL, benign keratinocytic lesion; DF, dermatofibroma; EC, expert consensus; FU, follow‐up; MEL, melanoma; NV, nevus; OTH‐b, other benign lesion; OTH‐m, other malignant lesion; PDS, pleomorphic dermal sarcoma; SCC, squamous cell carcinoma; VASC, benign vascular neoplasms including angioma and pyogenic granuloma

### Statistical analysis

Primary outcomes included sensitivity, specificity, and ROC‐AUC for binary classification of close‐up versus dermoscopic images. Descriptive statistics were reported using frequencies, means, and standard deviations. Two‐sided McNemar tests were conducted for pairwise comparison of sensitivities and specificities.^27^ The ROC‐AUCs for close‐up and dermoscopic images were statistically compared.^28^ Statistical significance was defined as *p* < 0.05. Analyses were performed with SPSS Version 25 (IBM, SPSS; Chicago, IL, USA).

## RESULTS

### Characteristics of patients and imaged lesions

The median age (± standard deviation (SD)) of patients was 64.0 (± 21.9) years (range: 1–94 years), 60.3% were male. Benign lesions (n = 155) included the diagnostic classes: BKL (n = 27, 17.4%), DF (n = 6, 3.9%), NV (n = 77, 49.7%), VASC (n = 27, 17.4%) and OTH‐b (n = 18, 11.6%) (Table 1). Malignant lesions (n = 195) included the diagnostic classes: AKIEC (n = 15, 7.7%), BCC (n = 35, 18.0%), MEL (n = 106, 54.4%), SCC (n = 6, 3.1%) and OTH‐m (n = 33, 16.9%) (Table 1). Most benign and malignant lesions were localized on the trunk (41.3% and 44.6%, respectively).

### Diagnostic accuracy of the DL‐CNN in clinical close‐up versus dermoscopic images

**FIGURE 2:**
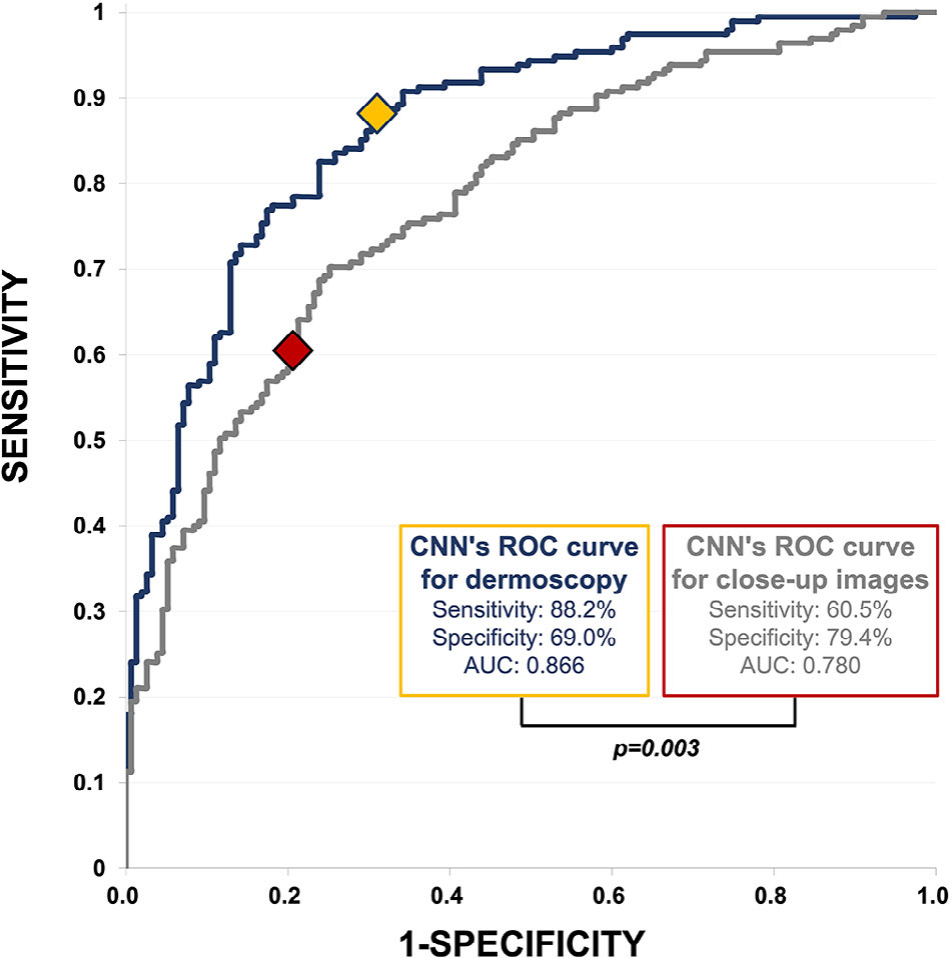
ROC curve of the DL‐CNN for dermoscopy (blue curve) versus close‐up images (gray curve) in the binary classification of benign versus malignant lesions. The DL‐CNN operating point for dermoscopy is indicated in yellow, and for close‐up images in red. Statistical analysis demonstrated a significantly higher diagnostic accuracy for the DL‐CNN ROC curve with dermoscopic images compared to clinical close‐up images (p = 0.003).

Boxplots in Figure 1 display the distribution of the malignancy scores assigned by the DL‐CNN (0–1, > 0.5 indicating malignancy) for benign (Figure 1b) and malignant (Figure 1d) lesions, categorized into more specific diagnostic categories. In general, the DL‐CNN scores for close‐up images were markedly lower than for dermoscopic images in both benign and malignant lesions (left boxplots in Figure 1b, d), with the exception of DF and VASC among benign and BCC among malignant lesions. When challenged with dermoscopic images the DL‐CNN demonstrated a sensitivity (95% confidence interval [CI]) of 88.2% (82.9%–92.0%), specificity of 69.0% (61.4%‐75.8%), and ROC‐AUC of 0.866 (0.860–0.873). In contrast, when challenged with close‐up images the sensitivity of DL‐CNN decreased significantly by approximately 30% (60.5% (53.5%–67.1%), *p* < 0.001), whereas the specificity increased by 10% (79.4% (72.3%–85.0%), *p* = 0.027). Overall, this resulted in a significantly reduced ROC‐AUC of 0.780 (0.772–0.790) for clinical close‐up images (p = 0.003) (Figure 2). Sensitivity and specificity for each lesion class are presented in online supplementary Table S1. To further characterize the diagnostic performance, we added sensitivity and specificity analyses for pigmented lesions by image type. Sensitivity was higher for dermoscopic images (92.5% (85.9%–96.2%)) than for close‐ups (65.6% (54.1%–72.1%)), while specificity was slightly higher for close‐ups (84.8% (75.3%–91.1%) vs. 78.5% (68.2%–86.1%)), reconfirming the overall performance pattern. To further evaluate the effect of threshold adjustment, we compared the DL‐CNN´s performance on clinical images using the predefined threshold (0.5) and the Youden‐optimized threshold (0.310). Sensitivity significantly increased from 60.5% to 69.7% (63.0%–75.8%, *p* < 0.001), while specificity decreased from 79.4% to 74.8% (67.5%–81.0%, *p* = 0.008). These findings indicate that optimizing the threshold leads to a more balanced classification, trading a moderate reduction in specificity for a clinically relevant gain in sensitivity, which may help reduce false‐negative diagnoses in practice. Compared to dermoscopic images, sensitivity remained significantly lower (69.7% vs. 88.2%, *p* < 0.001), while specificity showed no significant difference (74.8% vs. 69.0%, p = 0.19).

### Analyses of misclassified skin lesions: close‐up versus dermoscopic images

**FIGURE 3:**
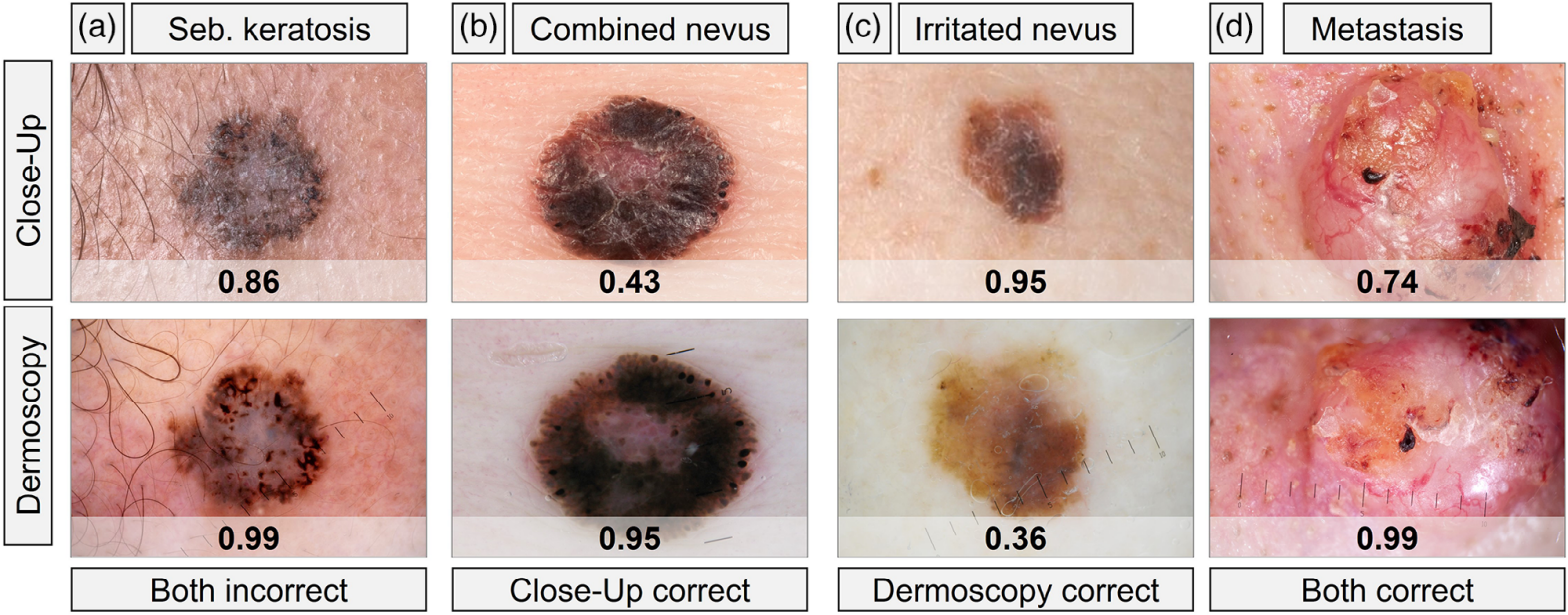
Representative lesions illustrating concordant and discordant DL‐CNN performance across clinical and dermoscopic images. Each row displays matched clinical and dermoscopic images of the same lesion and the DL‐CNN malignancy predictions. (a) Incorrect predictions on both modalities (such as seborrheic keratosis). (b) Correct prediction on the clinical image only (such as combined nevus with compound and Spitz nevi). (c) Correct prediction on the dermoscopic image only (such as mechanically irritated nevus). (d) Correct predictions on both modalities (such as cutaneous metastasis from breast cancer).

Certain underrepresented lesion types in the training set showed disproportionately greater decreases in accuracy. Performance by class (such as nevi and dermatofibroma) is detailed in Table 2. The number of correct DL‐CNN classifications was higher for dermoscopic images (279/350, 79.7%) than for close‐up images (241/350, 68.9%). It was correct for both modalities in 203 cases, incorrect for both in 33, correct only for dermoscopy in 76, and correct only for clinical images in 38 (Figure 3).

**TABLE 2:** Misclassified skin lesions.

Misclassified lesions		Close‐Up		Dermoscopy	
		n/n	(%)	n/n	(%)
*False negative classifications*	*Overall false negative rate*	77/195	(39.5)	23/195	(11.8)
**AKIEC**		5/15	33.3	3/15	20
Actinic Keratosis		2/5	40.0	2/3	66.7
Bowen Disease		3/5	60.0	1/3	33.3
**BCC**		13/35	37.1	1/35	2.9
Fibroepithelioma of Pinkus		1/13	0.1	–	–
BCC		12/13	92.3	1/35	2.9
**MEL**		38/106	35.9	8/106	7.5
Lentigo maligna		5/38	13.2	1/8	12.5
Melanoma in situ		3/38	7.9	2/8	25
Acrolentiginous melanoma		1/38	2.6	2/8	25
Amelanotic melanoma		1/38	2.6	–	–
Lentigo maligna melanoma		3/38	7.9	1/8	12.5
Nodular melanoma		3/38	7.9	–	–
Spitzoid melanoma		2/38	5.3	1/8	12.5
Superficial spreading melanoma		11/38	28.9	–	–
Melanoma. not specified		9/38	23.7	1/8	12.5
**SCC**		3/6	50	1/6	16.7
Keratoacanthoma		1/3	33.3	1/1	100
SCC		1/3	33.3	–	–
Bowen's carcinoma		1/3	33.3	–	–
**OTH‐m**		18/33	54.6	10/33	30.3
AFX		1/18	5.6	–	–
PDS		2/18	11.1	–	–
Metastasis of pulmonary carcinoma		2/18	11.1	–	–
Melanoma metastasis		10/18	55.6	10/10	100
Merkel cell carcinoma		2/18	11.1	–	–
Kaposi's sarcoma		1/18	5.6	–	–

False positive classifications	Overall false positive rate	32/155	(20.7)	48/155	(30.1)
**BKL**		5/32	15.6	10/32	31.3
Solar lentigo		2/5	40	–	–
Seborrheic keratosis		3/5	60	10/10	100
**DF**		3/6	50	2/6	33.3
**NV**		10/77	13	17/77	22.1
Dysplastic nevus		2/10	20	7/17	41.2
Congenital nevus		2/10	20	3/17	17.7
Blue nevus		2/10	20	1/17	5.9
Papillomatous nevus		1/10	10	3/17	17.7
Spitz nevus		2/10	20	2/17	11.7
Recurrence nevus		1/10	10	1/17	5.9
**VASC**		7/27	25.9	13/27	48.1
Angiokeratoma		1/7	14.3	1/13	7.7
Granuloma pyogenicum		1/7	14.3	1/13	7.7
Angiofibroma		1/7	14.3	1/13	7.7
Hemangioma		4/7	57.1	7/13	53.9
**OTH‐b**		7/18	38.9	6/18	33.3
Poroid Hidradenoma		1/7	14.3	–	–
Trichoblastoma		–	–	1/6	16.7
Zylindroma		1/7	14.3	1/6	16.7
Spiradenoma		–	–	1/6	16.7
Sebaceous adenoma		4/7	57.1	2/6	33.3
Cutaneous pseudolymphoma		–	–	1/6	16.7
Clear cell akanthoma		1/7	14.3	–	–

*Abbr*.: AFX, atypical fibroxanthoma; AKIEC, actinic keratosis/Bowen disease; BCC, basal cell carcinoma; BKL, benign keratinocytic lesion; DF, dermatofibroma; MEL, melanoma; NV, nevus; OTH‐b, other benign lesion; OTH‐m, other malignant lesion; PDS, pleomorphic dermal sarcoma; SCC, squamous cell carcinoma; VASC, benign vascular neoplasms including angioma and pyogenic granuloma

In dermoscopic images, a higher rate of false positives (FP) was observed, with 48 of 155 (31.0%) compared to 32 of 155 (20.7%) in close‐up images. In detail, nearly half of VASC (13 of 27, 48.2%) and over a third of BKL (10 of 32, 37.1%) were misclassified as malignant lesions in dermoscopic images (Table 2).

In close‐up images, false negatives (FN) were more frequently observed (77 of 195, 39.5%) than in dermoscopy (23 of 195, 11.8%). In detail, the DL‐CNN misclassified many SCC (3 of 6, 50.0%) and also OTH‐m lesions (18 of 33, 54.6%) in close‐up images (Table 2). Misclassified lesions in the category ”OTH‐m” included melanoma metastases (10 of 18, 55.6%), cutaneous metastases of pulmonary carcinoma (2 of 18, 11.1%), Merkel cell carcinoma (MCC) (2 of 18, 11.1%), pleomorphic dermal sarcoma (PDS) (2 of 18, 11.1%), atypical fibroxanthoma (AFX) (1 of 18, 5.6%), and Kaposi's sarcoma (1 of 18, 5.6%).

Overall, the diagnostic class of melanoma metastases was the most frequently misclassified subcategory within the OTH‐m category, for both clinical close‐up and dermoscopic images.

When investigating misclassifications of melanocytic lesions, we found that the DL‐CNN misclassified fewer melanomas in dermoscopic images (8 of 106, 7.6%) than in close‐up images (38 of 106, 35.9%). In contrast, fewer nevi were misclassified as malignant in close‐up images (10 of 77, 13.0%) compared to dermoscopy (17 of 77, 22.1%).

Excluding close‐up images taken by smartphones (39 of 350, 11.1%) slightly reduced the sensitivity and specificity of the DL‐CNN for digital single‐lens reflex camera (DSLR) close‐up images to 59.9% (52.4%–66.9%) and 78.4% (70.9%–84.4%), respectively. In contrast, analyzing the diagnostic accuracy of the DL‐CNN for smartphone‐captured close‐up images only resulted in a sensitivity of 65.2% (44.9%–81.2%) and a specificity of 100% (78.5%–100.0%).

## DISCUSSION

The development of DL‐CNNs for skin cancer diagnosis has been driven by the concept of a support‐tool capable of improving the diagnostic accuracy of clinicians.^8^, ^25^ Previous studies have indicated that greater benefits may be expected when less trained and less experienced clinicians, that is, non‐specialists, collaborate with a DL‐CNN.^8^ Most use cases for DL‐CNN support are therefore expected to be within clinical settings of primary care, teledermatology, and patient self‐examination via mobile phone‐based applications.^6^ Obviously, access to dermatoscopes with digital imaging is limited in non‐specialist settings, whereas close‐up images can be easily captured with smartphones or digital cameras. In addition, evolving devices for automated total body photography and 3D whole‐body skin imaging are currently being developed that will use artificial intelligence‐based models for classification of skin lesions in clinical images.^29^

Our study provides a critical head‐to‐head comparison of the diagnostic performance of a market‐approved DL‐CNN trained on dermoscopic images when challenged with close‐up images of the same skin lesions. The DL‐CNN´s ROC‐AUC for classifying dermoscopic images was significantly higher than that for close‐up images, reflecting superior overall diagnostic accuracy. False negatives were more frequently observed in close‐up images (39.5%) compared to dermoscopic images (11.8%), which is concerning given the high misclassification rates for certain malignant lesions such as SCC and other rare skin malignancies (OTH‐m), including melanoma metastases. This underscores the limitations of relying solely on clinical images for skin cancer screening, particularly in high‐risk individuals.

Interestingly, for professionally captured smartphone images, the DL‐CNN reached a specificity of 100%. Despite the limited sample size, this may suggest a systematic shift toward lower malignancy scores in lower‐quality images, potentially resulting in even more missed diagnoses and delayed treatment.^22^ While sensitivity was marginally higher in smartphone images than in DSLR images (65.2% vs. 59.9%), the marked trade‐off with specificity further underscores the risk of false negatives. These findings highlight that diagnostic accuracy may vary depending on the imaging device, even under professionally controlled conditions, emphasizing the need for standardized image quality protocols and patient guidance, especially in teledermatology.

Previously, a number of DL‐CNN models trained on dermoscopic images have demonstrated remarkable classification performance within this domain for melanoma diagnosis and also for other forms of skin cancer, even outperforming dermatologists.^9^, ^10^, ^11^, ^12^, ^25^ In contrast, there are only a few studies investigating clinical images for training of DL‐CNN models in skin cancer detection. In 2020 Birkenfeld et al. reported an accuracy of 75.9% for identifying suspicious pigmented skin lesions in wide‐field clinical images, although these authors had designed a multistep system rather than an end‐to‐end model.^30^ Other proof‐of‐principle studies evaluating DL‐CNN models using clinical images for melanoma versus benign nevi diagnosis have yielded variable results. In 2016 Nasr‐Esfahani and colleagues reported a sensitivity of 81% and a specificity of 80%,^31^ whereas in 2018 Han et al. found a sensitivity of 91% and a specificity of 90%.^32^ These discrepancies between studies may arise from the diversity of datasets and the heterogeneity of the methodologies employed.

In a recent study by Brinker et al. a DL‐CNN solely trained by dermoscopic images of melanomas and nevi was compared with the diagnostic performance of 145 dermatologists in a test set of 100 clinical images.^11^ Although challenged with a different target domain the DL‐CNN attained a sensitivity of 89.4% and specificity of 68.2%, which was similar to that of dermatologists. In contrast to the data of Brinker et al., we used a much broader range of diagnoses including rare subtypes in training and testing, and we provide a direct head‐to‐head comparison of clinical and dermoscopic images of the same lesions to better determine any loss or gain in accuracy. In another important study, Tschandl et al.^33^ focused on the diagnostic accuracy of a combined CNN (cCNN) trained with dermoscopic and close‐up images of non‐pigmented skin lesions. Again, their research demonstrated that the CNN performed on par with 95 human clinicians. In line with our data, the dermoscopic CNN was superior at diagnosing malignant cases, whereas the clinical close‐up CNN was superior at diagnosing benign cases.^33^ Moreover, the DL‐CNN in our study revealed lower diagnostic accuracy for rare skin lesions, particularly in close‐up images, suggesting a blind spot due to the underrepresentation of such cases in the training data.^17^, ^18^

In a more systematic approach, Rios‐Duarte et al. compared the classification performance (melanoma versus benign nevi) of three DL‐CNN models either trained by clinical images, dermoscopic images, or paired clinical and dermoscopic images of the same lesions.^34^ All three models were challenged with a test set comprising the same 119 cases with paired clinical and dermoscopic images. Interestingly, the CNN model solely trained with dermoscopic images showed the highest ROC‐AUC of 0.869 closely followed by the model trained with paired clinical and dermoscopic images (ROC‐AUC 0.822). Both aforementioned models that included dermoscopic images for training significantly outperformed the model solely relying on training with clinical images (ROC‐AUC 0.661, *p* < 0.001).^34^ Our data confirm this observation: melanomas were less frequently misclassified in dermoscopic images than in clinical close‐up images (7.6% vs. 35.9%), whereas fewer nevi were misclassified as malignant in clinical close‐up images (13.0% vs. 22.1% in dermoscopy). These findings confirm that while dermoscopy is essential for detecting melanomas, clinical close‐up images may promote a more conservative management approach in low‐risk individuals.

Taken together, these data indicate that training DL‐CNNs on feature‐rich and fine‐grained image domains such as microscopic and dermoscopic images is key to achieving robust diagnostic performance. Such precise, learned feature representations can later be deployed more successfully to feature‐poor target domains. In contrast, this observation does not apply for the reverse approach, with a feature‐poor source domain for training and a feature‐rich target domain for testing. In our view, it is worth further exploring the 10% gain in correct diagnoses when models were challenged with close‐up images of benign lesions, as observed in our study and by others. This could be implemented by fusion of DL‐CNN ensembles. Moreover, the 30% loss in sensitivity observed in our study could potentially be mitigated by applying transfer learning, specifically through fine‐tuning the DL‐CNN on clinical close‐up images of skin lesions. Finally, our threshold optimization analysis supports the importance of model calibration for specific clinical settings.^20^ Adjusting the decision threshold resulted in a balanced trade‐off between sensitivity and specificity, favoring a clinically meaningful gain in sensitivity. This suggests that careful threshold tuning may further enhance diagnostic safety by reducing false negatives, particularly relevant when close‐up images are the only modality available.

A potential limitation of our study may be the lack of dermatologists as a performance benchmark, as the focus was to use the diagnostic accuracy of the DL‐CNN for dermoscopic images as the reference standard. Additional limitations arose from the predominance of light‐skinned patients, as dermoscopic features of certain diagnoses may vary by skin type,^35^ reflecting the patient population at our institution. Another limitation is the use of a convenience sample and its implications for a reduced generalizability. Furthermore, the study did not include metadata for the diagnostic classification by the DL‐CNN, such as age, anatomical site, and lesion history. However, this limitation was knowingly accepted, as current market‐approved DL‐CNNs for skin lesion classification are intended to support screening rather than provide management recommendations.^8^ The study intentionally included a high proportion of malignant lesions to ensure histologically confirmed cases as the diagnostic gold standard. Besides, it is worth noting that differences between dermoscopic and close‐up images of the same lesion are not solely caused by acquisition techniques but could also arise from scaling, rotation, and artefacts. To address these issues, standardized preprocessing was applied to minimize potential distribution shifts that could impact CNN performance.^14^, ^15^, ^16^, ^19^ Lastly, generalizability of our findings may be limited by using images from a single institution and a specific model architecture. Nonetheless, our study remains relevant by focusing on market‐approved DL‐CNNs designed for clinical use, as these models represent the practical tools currently employed in clinical settings. In contrast, state‐of‐the‐art research architectures, as discussed by Minderer et al., may demonstrate greater robustness to distribution shifts.^19^

Our findings reveal that a DL‐CNN trained on the feature‐rich domain of dermoscopic images experiences a 30% drop in sensitivity and a 10% gain in specificity when applied to the feature‐poor target domain of clinical close‐up images. Earlier studies have shown that combining clinical and dermoscopic images during training yields only marginal sensitivity improvements compared to training exclusively on dermoscopic images. This suggests that DL‐CNNs are generally less effective when applied solely to clinical images. This has important implications for DL‐CNN models in teledermatology, smartphone‐based applications, and whole‐body skin imaging devices. Clinical images, with their high specificity, may be effective for triaging clearly benign lesions. However, for lesions with uncertain malignant potential, achieving a higher diagnostic standard will likely require dermoscopic images. Thus, ensuring the availability of affordable digital dermoscopy devices in non‐specialist settings is critical. Looking ahead, the evolution of DL‐CNNs may favor more versatile foundation models such as Vision Transformers or hybrid CNN‐transformer models that can be fine‐tuned for specific tasks.^20^, ^21^ A key challenge remains: how much fine‐tuning is necessary to enhance performance with clinical close‐up images? Comparative studies between DL‐CNNs, vision transformers (ViTs), and hybrid models could provide deeper insights.

## FUNDING

Katharina Susanne Kommoss is funded by the Physician‐Scientist Program of Heidelberg University, Faculty of Medicine.

## CONFLICT OF INTEREST STATEMENT

H.A. Haenssle received honoraria and/or travel expenses from companies involved in the development of devices for skin cancer screening: Scibase AB, FotoFinder Systems GmbH, Heine Optotechnik GmbH, Magnosco GmbH. J.K. Winkler received honoraria from FotoFinder Systems GmbH. W. Stolz received honoraria from FotoFinder Systems GmbH and Heine Optotechnik GmbH. The other authors state no conflict of interest related to the study.

## Supporting information



Supplementary information

Supplementary information

Supplementary information

Supplementary information
