# Supplementary material for: Robustness of a convolutional neural network trained on dermoscopic images and challenged with close‐up images
Source: J Dtsch Dermatol Ges. 2025 Oct 11;24(4):504–13. doi: 10.1111/ddg.15900 (PMC13059056; doi:10.1111/ddg.15900)
Supplement: Supplementary file 1 — Supplementary information [file DDG-24-504-s001.docx]

**Anhang M1. METHODEN**

**Architektur des *Convolutional Neural Networks* (CNN)**

Die Architektur und Trainingsmethoden des in dieser Studie eingesetzten CNNs wurden bereits veröffentlicht.¹ Die Prototypversion wurde weiterentwickelt, diese hat kürzlich eine Marktzulassung für Europa erhalten. Sie basiert auf Googles Inception_v4-CNN-Architektur, wurde auf dem ImageNet-Datensatz (1,28 Millionen Bilder in 1.000 Objektklassen) vortrainiert und besteht aus 27 Schichten.² Die Inception_v4-Architektur ist bekannt für ihre hohe Vorhersagegenauigkeit. Sie ist eine reine Inception-Variante ohne Residualverbindungen und erreicht eine ähnliche Erkennungsleistung wie Inception-ResNet-v2. Das Netzwerk beginnt mit dem Input eines Bildes mit einer Auflösung von 299×299 Pixeln, das an das „Stamm-Modul“ mit drei Inception-Modulen weitergeleitet wird. Das Ergebnis wird viermal durch das „Inception-A“-Modul geführt, das aus einer Kombination von Average Pooling, 1×1- und 3×3-Convolutions besteht. Der zusammengeführte Output durchläuft ein Reduktionsmodul, bevor er siebenmal in das „Inception-B“-Modul eingespeist wird, das aus Average Pooling, 1×1-, 7×1- und 1×7-Convolutions besteht. Anschließend wird das Ergebnis erneut durch ein Reduktionsmodul geleitet, gefolgt vom dreifachen Durchlauf durch das „Inception-C“-Modul, das Average Pooling, 1×1-, 1×3- und 3×1-Convolutions kombiniert. Abschließend erfolgt eine durchschnittliche Pooling-Schicht, eine Dropout-Schicht und die Klassifikation über eine Softmax-Schicht, die die finale diagnostische Einordnung ausgibt.

**Datenerhebung**

Dermatoskopische Bilder sowie die zugehörigen Diagnose-Labels von Läsionen unterschiedlicher Subtypen und anatomischer Lokalisationen wurden aus mehreren Quellen gesammelt – darunter Beiträge von über 50 kooperierenden Dermatolog:innen weltweit sowie öffentlich zugängliche Bilder aus dem ISIC-Dermatoskopiearchiv (International Skin Imaging Collaboration).³ Für das Training des CNN wurden insgesamt 129.487 dermatoskopische Bilder mit zugehörigen Diagnose-Labels verwendet, davon 100.021 benigne und 29.466 maligne Läsionen. Die benignen Läsionen umfassten 64.536 Nävi, 6.631 benigne Keratosen, 1.076 vaskuläre Läsionen, 864 Dermatofibrome und 26.914 Läsionen mit sonstigen Diagnosen. Die malignen Läsionen bestanden aus 11.826 Melanomen, 11.232 Basalzellkarzinomen, 1.978 aktinischen Keratosen, 596 Plattenepithelkarzinomen und 3.834 weiteren malignen Läsionen. Die Diagnose-Labels wurden entweder histopathologisch gesichert oder – bei nicht-exzidierten Läsionen – durch erfahrene Dermatolog:innen oder unauffällige serielle dermatoskopische Verlaufskontrollen bestätigt. Jeder Datensatz wurde im Verhältnis 9:1 in Trainings- und Validierungs-/Testdaten unterteilt. Zusätzlich wurden manuell validierte Bilddatensätze mit diagnostisch schwierigen Fällen (aus menschlicher Sicht) für eine differenzierte Bewertung der Modellleistung erstellt.

**Datenvorverarbeitung**

Aufgrund des Ungleichgewichts zwischen den häufigeren benignen und selteneren malignen Läsionen war ein Oversampling erforderlich. Dieses erfolgte über verschiedene geometrische Bildtransformationen (z. B. zufällige Helligkeit, Spiegelung, Rotation, minimale Farbverzerrung), die die ursprüngliche Klassenzugehörigkeit der Bilder nicht veränderten. In unserem Modell wurden ein oder mehrere dieser Transformationen zufällig (basierend auf einer Normalverteilung) auf Bilder des ursprünglichen Datensatzes angewendet, um ein Verhältnis von 1:1 zwischen benignen und malignen Läsionen zu erreichen. Für jede Epoche wurden Bilder zufällig aus den Batches ausgewählt und ggf. mit Transformationen versehen. Dadurch wurden dieselben Originaldaten in leicht veränderter Form über mehrere Trainingsdurchläufe hinweg erneut eingespeist.

**Trainingsprozess**

Das CNN wurde mittels Transfer Learning auf der Grundlage von Googles Inception_v4-Architektur initialisiert.⁴ Dabei wurden die Gewichte der Neuronen nicht zufällig gesetzt, sondern entsprechend dem vortrainierten Modell übernommen, das auf einer Vielzahl unterschiedlicher Objektklassen basiert. Diese Herangehensweise ermöglichte ein effektives Training mit dem spezifischeren Datensatz dermatoskopischer Bilder, verkürzte die Trainingszeit, verbesserte die Leistung und verringerte das Risiko eines lokalen Minimums beim Gradientenabstieg. Da die Netzwerkarchitektur festgelegte Bildgrößen verlangt, wurden Bilder aus unterschiedlichsten Aufnahmegeräten durch Zuschneiden und Auffüllen auf eine Auflösung von 299×299 Pixel skaliert. Das Training mit dem spezifischen Datensatz erfolgte unter Verwendung des AdaM-Optimierers⁵ (Adaptive Moment Estimation), einer Variante des stochastischen Gradientenabstiegs, und mit Googles Deep-Learning-Bibliothek TensorFlow.⁶ Die Lernrate war auf 0,001 festgelegt, mit einem Abklingfaktor von 0,9, einem Momentum von 0,999 und einem ϵ-Wert von 10⁻⁸. Die Batchgröße betrug 16, und die Anzahl der Epochen lag bei 80. Während des Trainings wurden keine „Early Stopping“-Kriterien verwendet.

**Referenzen**

1. Haenssle HA, Fink C, Schneiderbauer R, Toberer F, Buhl T, Blum A et al. Man against machine: diagnostic performance of a deep learning convolutional neural network for dermoscopic melanoma recognition in comparison to 58 dermatologists. Ann Oncol 2018;29:1836-42.

2. Szegedy C, Vanhoucke V, Ioffe S *et al.* Rethinking the inception architecture for computer

vision. In: *Proceedings of the IEEE conference on computer vision and pattern recognition*.

2016; 2818-26.

3. Finnane A, Curiel-Lewandrowski C, Wimberley G *et al.* Proposed Technical Guidelines for

the Acquisition of Clinical Images of Skin-Related Conditions. *JAMA dermatology* 2017;

**153**: 453-7.

4. Pan SJ, Yang Q. A survey on transfer learning. *IEEE Transactions on knowledge and data*

*engineering* 2010; **22**: 1345-59.

5. Ruder S. An overview of gradient descent optimization algorithms. *arXiv preprint*

*arXiv:1609.04747* 2016.

6. Abadi M, Barham P, Chen J *et al.* Tensorflow: A system for large-scale machine learning. In: *12th {USENIX} Symposium on Operating Systems Design and Implementation ({OSDI} 16)*. 2016; 265-83.

**Supplementary Figure S1**

The global Inception_v4 network architecture
