# Supplementary material for: Robustness of a convolutional neural network trained on dermoscopic images and challenged with close‐up images
Source: J Dtsch Dermatol Ges. 2025 Oct 11;24(4):504–13. doi: 10.1111/ddg.15900 (PMC13059056; doi:10.1111/ddg.15900)
Supplement: Supplementary file 2 — Supplementary information [file DDG-24-504-s002.docx]

**Appendix M1. METHODS**

**Convolutional neural network architecture**

The architecture and training methods related to the CNN prototype used in this study were previously published.^1^ We further developed the prototype version that recently received market-approval for Europe and which is based on Google’s Inception_v4 CNN architecture, pretrained on the ImageNet dataset (1.28 million images across 1,000 object classes) and composed of 27 layers.^2^ Known by its high predictive performance, Inception_v4 architecture is a pure Inception variant without residual connections that has roughly the same recognition performance as Inception-ResNet-v2. The network starts with the input of a 299x299 pixels image that is fed to the stem module containing 3 inception modules. The output is fed to the ‘Inception A’ module four times. ‘Inception A’ contains combination of average pooling, 1x1 convolutions and 3x3 convolutions. The concatenated output is run through a reduction module. Next, the data is fed to the ‘Inception B’ module seven times. The ‘Inception B’ module is the concatenation of average pooling, 1x1 convolutions and 7x1 and 1x7 convolutions. Resulting data is fed to another reduction module, followed by an ‘Inception C’ module three times. ‘Inception C’ is composed of average pooling, 1x1 convolution, and 1x3 and 3x1 convolutions. The result is run through an average pooling and a dropout layer and finally through the Softmax layer that puts out the final diagnostic classification.

**Data collection**

Dermoscopic images and corresponding diagnostic labels of lesions of different subtypes and anatomic localizations were collected from multiple sources, including more than 50 cooperating dermatologists around the globe and publicly available images from the International Skin Imaging Collaboration (ISIC) dermoscopic archive.^3^ For CNN training a total of 129.487 dermoscopic images with corresponding diagnostic labels were used comprising 100.021 benign and 29.466 malignant lesions. Benign lesions included 64536 nevi, 6631 benign keratoses, 1076 vascular lesions, 864 dermatofibromas and 26914 lesions with other diagnoses. Malignant lesions comprised 11826 melanomas, 11232 basal cell carcinomas, 1978 actinic keratoses, 596 squamous cell carcinomas and 3834 lesions with other diagnoses. Diagnostic labels were validated by histopathology, or, in case of non-excised lesions, by experienced dermatologists and/or inconspicuous sequential digital dermoscopy follow-up examinations. Each collected dataset was split at a 9:1 ratio for training and validation/testing. Additional image sets for validation and testing were manually compiled and contained difficult-to-diagnoses cases (from a human perspective) to determine the model’s performance.

**Data pre-processing**

Because of imbalances in the ratio of (more frequent) benign and (less frequent) malignant lesions, an oversampling of the data was needed. We used an oversampling by several geometric image transformations (e.g. random brightness, reflection, rotation, minimal color distortion) which left the original classification unchanged. In our model, we randomly applied (using a normal distribution) one or more of these transformations on images of the original dataset to finally attain a ratio of 1:1 for images of benign versus malignant lesions. For each epoch, images were randomly selected from the batches, and none, one, or several of the transformations were applied. As a result, the same original data with slight variations were re-entered from one epoch to the other.

**Training Process**

We initialized Google’s Inception_v4 CNN by using transfer learning,^4^ i.e. the neurons’ weights were kept as previously set in the model pretrained across multiple object classes, instead of using random initiation. By using this approach, we were able to finish the training with the limited specific dataset (dataset of dermoscopic images). This process allowed us to shorten the training time as well as improve the results and minimizes the risk of the gradient descent to get blocked in a local minimum. Due to the design of the network, input images derived from a range of different capturing devices had to be resized to 299x299 pixels by padding and cropping. Training with the limited specific dataset was attained by using a variation of the stochastic gradient descent algorithm called Adaptive Moment estimation (AdaM) optimizer^5^ and Google’s deep learning library TensorFlow.^6^ A fixed learning rate at 0.001 with 0.9 decay factor, a momentum of 0.999 and ϵ = 10^-8^ were used. The training batch size was 16, and the number of epochs was 80. No early stopping criteria were used during all the training instances.

**Reference List**

1. Haenssle HA, Fink C, Schneiderbauer R, Toberer F, Buhl T, Blum A et al. Man against machine: diagnostic performance of a deep learning convolutional neural network for dermoscopic melanoma recognition in comparison to 58 dermatologists. Ann Oncol 2018;29:1836-42.

2. Szegedy C, Vanhoucke V, Ioffe S *et al.* Rethinking the inception architecture for computer

vision. In: *Proceedings of the IEEE conference on computer vision and pattern recognition*.

2016; 2818-26.

3. Finnane A, Curiel-Lewandrowski C, Wimberley G *et al.* Proposed Technical Guidelines for

the Acquisition of Clinical Images of Skin-Related Conditions. *JAMA dermatology* 2017;

**153**: 453-7.

4. Pan SJ, Yang Q. A survey on transfer learning. *IEEE Transactions on knowledge and data*

*engineering* 2010; **22**: 1345-59.

5. Ruder S. An overview of gradient descent optimization algorithms. *arXiv preprint*

*arXiv:1609.04747* 2016.

6. Abadi M, Barham P, Chen J *et al.* Tensorflow: A system for large-scale machine learning. In: *12th {USENIX} Symposium on Operating Systems Design and Implementation ({OSDI} 16)*. 2016; 265-83.

**Supplementary Figure S1**

The global Inception_v4 network architecture
