# Supplementary figures and images for: Robustness of a convolutional neural network trained on dermoscopic images and challenged with close‐up images
Source: J Dtsch Dermatol Ges. 2025 Oct 11;24(4):504–13. doi: 10.1111/ddg.15900 (PMC13059056; doi:10.1111/ddg.15900)

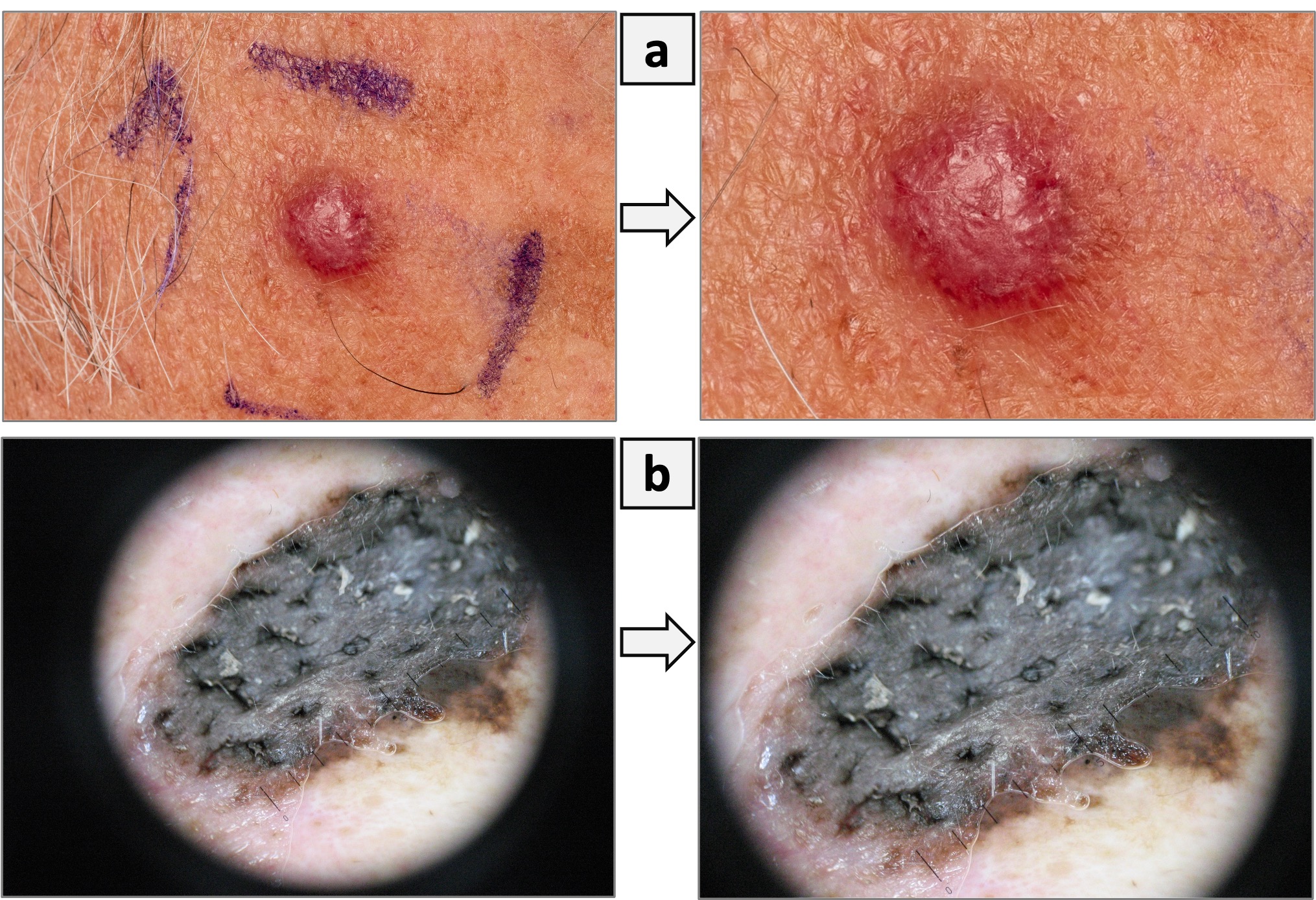

Supplement: Supplementary file 3 — Supplementary information [file DDG-24-504-s004.jpg]
