# Supplementary material for: Robustness of a convolutional neural network trained on dermoscopic images and challenged with close‐up images
Source: J Dtsch Dermatol Ges. 2025 Oct 11;24(4):504–13. doi: 10.1111/ddg.15900 (PMC13059056; doi:10.1111/ddg.15900)
Supplement: Supplementary file 4 — Supplementary information [file DDG-24-504-s003.docx]

**Supplementary Table 1. Sensitivity and specificity per lesion class.**

| **Sensitivity (%)** | **Close-Up** | **Dermoscopy** |
| --- | --- | --- |
|  |  |  |
| **AKIEC** | **66.7** | **80.0** |
| **BCC** | **62.9** | **97.1** |
| **MEL** | **64.1** | **92.5** |
| **SCC** | **50.0** | **83.3** |
| **OTH-m** | **45.4** | **69.7** |
| **Specificity (%)** |  |  |
| **BKL** | **84.4** | **68.7** |
| **DF** | **50.0** | **66.7** |
| **NV** | **87.0** | **77.9** |
| **VASC** | **74.1** | **51.9** |
| **OTH-b** | **61.1** | **66.7** |
